# Artificial intelligence in BreastScreen Norway: a retrospective analysis of a cancer-enriched sample including 1254 breast cancer cases

**DOI:** 10.1007/s00330-023-09461-y

**Published:** 2023-03-14

**Authors:** Henrik Wethe Koch, Marthe Larsen, Hauke Bartsch, Kathinka Dæhli Kurz, Solveig Hofvind

**Affiliations:** 1 Department of Radiology, Stavanger University Hospital, Stavanger, Norway; 2 Faculty of Health Sciences, University of Stavanger, Stavanger, Norway; 3 Section for Breast Cancer Screening, Cancer Registry of Norway, Oslo, Norway; 4 Department of Radiology, Haukeland University Hospital, Bergen, Norway; 5 Department of Electrical Engineering and Computer Science, Faculty of Science and Technology, The University of Stavanger, Stavanger, Norway; 6 Department of Health and Care Sciences, Faculty of Health Sciences, The Arctic University of Norway, Tromsø, Norway

**Keywords:** Mammography, Breast neoplasm, Mammographic density, Artificial intelligence, Mass screening

## Abstract

**Objectives:**

To compare results of selected performance measures in mammographic screening for an artificial intelligence (AI) system versus independent double reading by radiologists.

**Methods:**

In this retrospective study, we analyzed data from 949 screen-detected breast cancers, 305 interval cancers, and 13,646 negative examinations performed in BreastScreen Norway during the period from 2010 to 2018. An AI system scored the examinations from 1 to 10, based on the risk of malignancy. Results from the AI system were compared to screening results after independent double reading. AI score 10 was set as the threshold. The results were stratified by mammographic density.

**Results:**

A total of 92.7% of the screen-detected and 40.0% of the interval cancers had an AI score of 10. Among women with a negative screening outcome, 9.1% had an AI score of 10. For women with the highest breast density, the AI system scored 100% of the screen-detected cancers and 48.6% of the interval cancers with an AI score of 10, which resulted in a sensitivity of 80.9% for women with the highest breast density for the AI system, compared to 62.8% for independent double reading. For women with screen-detected cancers who had prior mammograms available, 41.9% had an AI score of 10 at the prior screening round.

**Conclusions:**

The high proportion of cancers with an AI score of 10 indicates a promising performance of the AI system, particularly for women with dense breasts. Results on prior mammograms with AI score 10 illustrate the potential for earlier detection of breast cancers by using AI in screen-reading.

**Key Points:**

*• The AI system scored 93% of the screen-detected cancers and 40% of the interval cancers with AI score 10.*

*• The AI system scored all screen-detected cancers and almost 50% of interval cancers among women with the highest breast density with AI score 10.*

*• About 40% of the screen-detected cancers had an AI score of 10 on the prior mammograms, indicating a potential for earlier detection by using AI in screen-reading.*

**Supplementary Information:**

The online version contains supplementary material available at 10.1007/s00330-023-09461-y.

## Introduction

Breast cancer is the most common cancer worldwide and the most common cause of cancer related death among women [1]. Early detection through systematic mammographic screening is shown to reduce the incidence of advanced disease and breast cancer mortality [2, 3].

In the current screening setting, the radiological accuracy is suboptimal, with a sensitivity below 80% in general and as low as 50% for women with mammographic dense breasts [3–7]. Some of the undetected cancers present as symptomatic interval cancers within the next screening round, and these cases are associated with less prognostic favorable histopathological tumor characteristics compared to screen-detected cancers [8, 9]. Retrospective consensus-based informed review studies have classified 20–30% of the interval cancers as well as screen-detected cancers as missed by the radiologists, based on prior screening mammograms [10–12].

With recent advances in machine learning and deep learning, artificial intelligence (AI) are attracting considerable attention as means to increase sensitivity of mammographic screening. However, knowledge about the AI systems performance, based on real screening data, is sparse and as far as we are aware, no studies have explored AI malignancy risk score of prior mammograms for screen-detected cancers. In addition, studies reporting histopathologic tumor characteristics for breast cancers with different AI malignancy risk scores and studies stratifying results by mammographic density are limited [13, 14].

A screening program using independent double reading, as in BreastScreen Norway, represents a substantial workload for the radiologists. However, the majority of the examinations, more than 96%, are without signs of breast cancer [15]. To potentially reduce time spent on interpreting negative screening mammograms, AI systems have been proposed as a tool to support or even replace radiologists in the reading process [16–22]. In a retrospective study simulating how an AI system could be used to triage and exclude selected examinations from the radiologist reading stream, the reader volume was reduced by 63% compared to standard double reading, without reducing cancer detection [14].

We took advantage of the data collected in Rogaland as a part of BreastScreen Norway and compared performance of an AI system to independent double reading. Performance was measured according to screening outcomes, including consensus, recall, cancer detection, and histopathological tumor characteristics. Further, we stratified the results by mammographic density and investigated the AI score on the examinations prior to the examination where the cancers were detected.

## Materials and methods

The data was disclosed with legal bases in the Cancer Registry of Norway Regulations § 3–5, and the retrospective study was approved by the Regional Committee for Medical and Health Research Ethics (2018/2574).

**Fig. 1:**
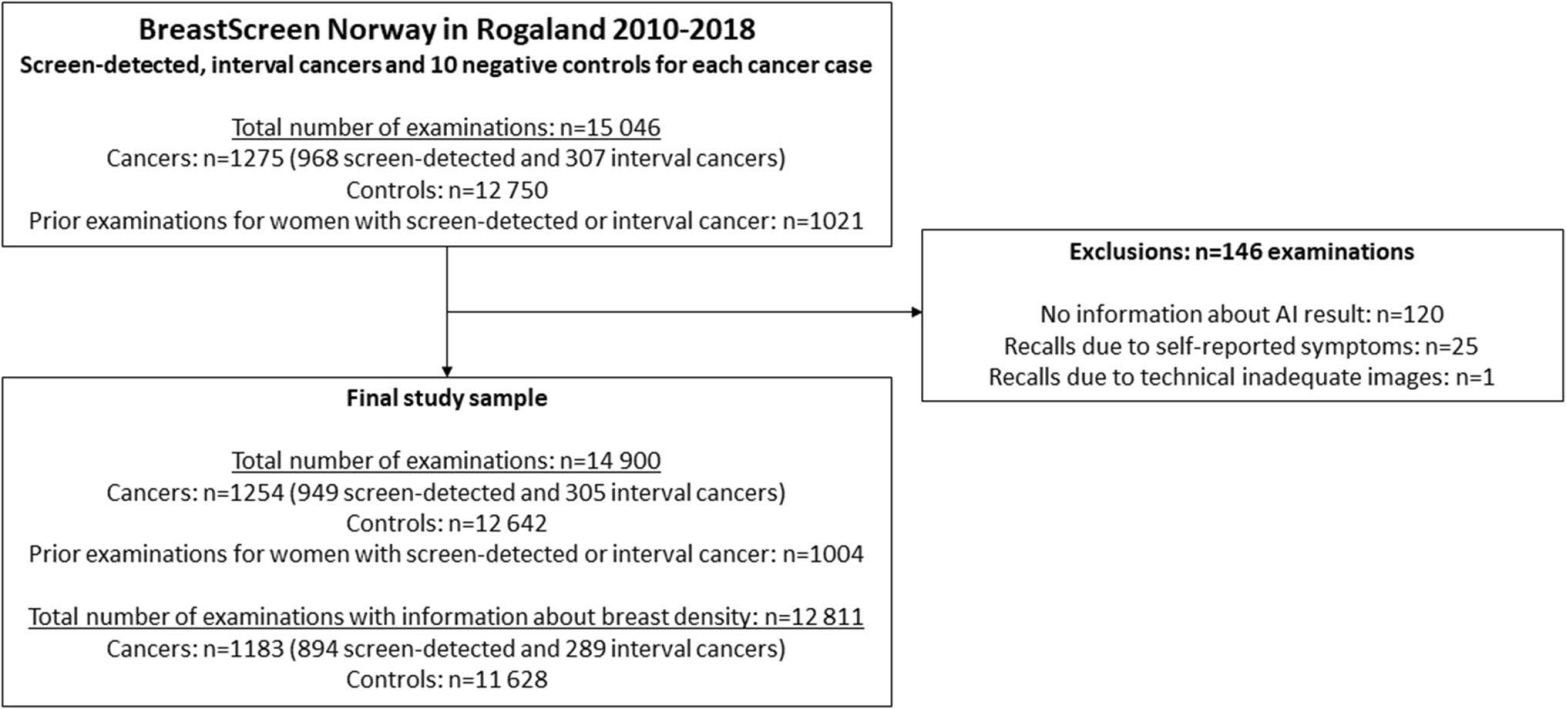
Flowchart of the study sample

All screen-detected and interval breast cancers detected at Stavanger University Hospital from 2010 to 2018 were identified from the Cancer Registry database and matched with 10 random negative examinations, by age at screening and year of screening examination. The data included 1275 cancer cases and 12,750 controls (Fig. 1). In addition, screening and AI information from prior screening examination for the cancer cases were included, if available (*n* = 1021). All mammograms were extracted from the Picture Archiving and Communication System and processed with the AI system. Results from the AI system were merged with pseudonymized screening information using random study identification numbers. After exclusions, digital mammograms of 14,900 examinations were left in the study population, including 1254 breast cancer cases, 12,642 negative controls, and prior examinations for 1004 women diagnosed with breast cancer. All women were screened with GE Senographe Essential. Screening interpretations were performed by 13 breast radiologists with a median annual reading volume of about 7000 readings and 1–22 years of experience with screening interpretation, including the study period.

BreastScreen Norway offers women aged 50–69 years biennial mammographic screening. Standard interpretation procedure is independent double reading, and each radiologist assigns a score from 1 to 5 of each breast to indicate suspicion of malignancy: 1, negative for malignancy; 2, probably benign; 3, intermediate suspicion of malignancy; 4, probably malignant; 5, high suspicion of malignancy. Examinations with an interpretation score of 2 or higher by either or both radiologists are discussed at a consensus meeting to determine recall or not [15].

### AI system

The AI system used in this study was Transpara version 1.7.0 (ScreenPoint Medical). This commercially available AI system is Conformité Européenne (CE) marked and cleared by the U.S. Food and Drug Administration (FDA). The AI system provides a continuous score from 0.0 to 10.0 (raw score) and categorized examinations into 10 groups based on the highest overall exam-level raw score. This is referred to as AI score and ranged from 1 to 10. An AI score of 1 indicated a low risk of abnormal findings and 10 indicated high risk. The AI system uses convolutional neural networks to analyze mammograms and has been trained on mammograms from different screening programs and mammograms from several vendors.

### Variables of interest

Recall was defined as screening examinations resulting in further assessments due to abnormal mammographic findings. Screen-detected cancer was defined as breast cancer diagnosed after a recall and within 6 months after the screening examination, and interval cancer was defined as breast cancers diagnosed within 24 months after a negative screening or 6–24 months after a false-positive screening result [15]. Both ductal carcinoma in situ (DCIS) and invasive carcinoma were considered breast cancer. Cancer type was classified as DCIS, invasive carcinoma of no special type, invasive lobular carcinoma, and other invasive. Based on lymph node status, tumor diameter, and histologic grade, the Nottingham Prognostic Index (NPI) was calculated and categorized as “Excellent, ≤ 2.4”, “Good, > 2.4 and ≤ 3.4”, “Moderate, > 3.4 and ≤ 5.4”, and “Poor, > 5.4” for invasive cancers [23]. Quantitative volumetric breast density, Volpara Density Grade (VDG; 1, 2, 3, 4), was obtained from an automated software (VolparaDensity, version 1.5.0; Volpara Health Technologies) [24]. The categorization is analogous to the four-category Breast Imaging Reporting and Data System, 5th edition classification system (a, b, c, d) [25].

### Statistical analysis

The performance of the AI system was evaluated descriptively by assessing the number and percentage of screen-detected cancers from independent double reading and interval cancers for each AI score. Categorical variables were presented with frequencies and percentages. Continuous variables were presented with mean and standard deviation (SD) or median and interquartile range (IQR) according to the distribution. Sensitivity for the AI system was compared to the sensitivity for the first reader and was calculated by setting the continuous AI raw score threshold value so that AI had the exact same specificity as the first reader. For the first reader, screen-detected and interval cancers not selected for consensus were considered false negatives. Screen-detected and interval cancers selected for consensus were considered true positives. This might also include recall and assessment with negative outcome, but with a later interval cancer. When calculating sensitivity for the AI system, all screen-detected and interval cancers with a raw score above the given threshold value were considered true positives. Workload reduction in a theoretical triage setting was calculated as the percentage of mammograms that would be excluded from the radiologist reading stream. The pre-screen-reading triage exclusion threshold was set to AI score ≤ 5, in accordance to relevant literature [14, 17, 19, 21]. All analyses were performed with Stata version 17.0 for Windows ((StataCorp).

## Results

Mean age for the women in the study sample was 58 years (SD = 6) and 25% of the women were prevalently screened in the program. 92.7% (880/949) of the screen-detected and 40.0% (122/305) of the interval cancers had an AI score of 10 (Table 1). In total, 79.9% (1002/1254) of all breast cancers in the study sample had an AI score of 10, and 2.1% (26/1254) had an AI score of 1. Among the 69 screen-detected cancers with an AI score below 10, 30.4% (21/69) were selected for consensus by only one of the two radiologists (Table 2). Specificity of the first reader was 97.6%. By setting the AI raw score threshold at equal specificity level, the sensitivity of the AI system was 68.3% compared to 68.6% for the first reader.

**Table 1:** Frequencies and percentages of 13,896 mammography examinations, examinations with a negative outcome, screen-detected cancers, interval cancers, and all cancers combined, stratified by malignancy score from the AI system (AI score)

AI score	All examinations	Negative outcome	Screen-detected cancer	Interval cancer	All cancers
1	3049 (21.9%)	3023 (23.9%)	3 (0.3%)	23 (7.5%)	26 (2.1%)
2	1072 (7.7%)	1060 (8.4%)	2 (0.2%)	10 (3.3%)	12 (1.0%)
3	1173 (8.4%)	1158 (9.2%)	1 (0.1%)	14 (4.6%)	15 (1.2%)
4	1113 (8.0%)	1096 (8.7%)	3 (0.3%)	14 (4.6%)	17 (1.4%)
5	1068 (7.7%)	1049 (8.3%)	6 (0.6%)	13 (4.3%)	19 (1.5%)
6	960 (6.9%)	939 (7.4%)	4 (0.4%)	17 (5.6%)	21 (1.7%)
7	961 (6.9%)	936 (7.4%)	7 (0.7%)	18 (5.9%)	25 (2.0%)
8	1107 (8.0%)	1073 (8.5%)	15 (1.6%)	19 (6.2%)	34 (2.7%)
9	1238 (8.9%)	1155 (9.1%)	28 (3.0%)	55 (18.0%)	83 (6.6%)
10	2155 (15.5%)	1153 (9.1%)	880 (92.7%)	122 (40.0%)	1002 (79.9%)
Total	13,896 (100%)	12,642 (100%)	949 (100%)	305 (100%)	1254 (100%)

**Table 2:** Frequencies and percentages of screening examinations discussed at consensus, recalled for further assessment, and screen-detected and interval cancers with a positive interpretation by one or both radiologists, stratified by malignancy score from the AI system (AI score)

AI score	Discussed at consensus	Recalled	Screen-detected cancers		Interval cancers		
			Positive interpretation by one radiologist	Positive interpretation by both radiologists	Recalled, negative outcome	Positive interpretation by one radiologist	Positive interpretation by both radiologists
1	71 (4.7%)	3 (0.3%)	1	2	0	0	0
2	26 (1.7%)	2 (0.2%)	1	1	0	0	1
3	36 (2.4%)	1 (0.1%)	0	1	0	1	0
4	51 (3.3%)	4 (0.4%)	1	2	1	2	0
5	51 (3.3%)	6 (0.6%)	2	4	0	0	0
6	49 (3.2%)	4 (0.4%)	1	3	0	2	0
7	56 (3.7%)	7 (0.7%)	1	6	0	2	0
8	61 (4.0%)	15 (1.6%)	3	12	0	0	0
9	114 (7.5%)	29 (3.0%)	11	17	1	9	1
10	1011 (66.3%)	887 (92.6%)	194	686	7	17	6
Total	1526 (100%)	958 (100%)	215	734	9	33	8

### Histopathological tumor characteristics

Among the screen-detected cancers with an AI score of 10, 79.1% (696/880) were invasive (Table 3). Median tumor diameter for these cases was 14 mm (IQR: 10–21), 28.9% (199/688) was histologic grade 3, and 21.5% (148/688) lymph node positive (Table 4). For those with an AI score below 10, 84.1% (58/69) was invasive, median diameter 11 mm (IQR: 8–15), 29.1% (16/55) histologic grade 3, and 17.9% (10/56) lymph node positive. Mean NPI was 3.6 (SD = 1.1) for cases with an AI score of 10 and 3.5 (SD = 1.0) for cases below 10.

**Table 3:** Histopathological tumor characteristics (frequencies, percentages, and AI sensitivity) of screen-detected and interval cancers with a malignancy score from the AI system (AI score) of 10 and AI score < 10

	Screen-detected cancers, *n* = 949			Interval cancers, *n* = 305		
	AI score = 10, *n* = 880	AI score < 10, *n* = 69	AI score = 10, sensitivity	AI score = 10, *n* = 122	AI score < 10, *n* = 183	AI score = 10, sensitivity
Ductal carcinoma in situ	184 (20.9%)	11 (15.9%)	94.4%	4 (3.3%)	6 (3.3%)	40.0%
Invasive carcinoma no special type	608 (69.1%)	49 (71.0%)	92.5%	99 (81.2%)	137 (74.9%)	41.9%
Invasive lobular carcinoma	57 (6.5%)	5 (7.3%)	91.9%	14 (11.5%)	34 (18.6%)	29.2%
Other invasive	31 (3.5%)	4 (5.8%)	88.6%	5 (4.1%)	6 (3.3%)	45.5%
Invasive tumors	696 (79.1%)	58 (84.1%)	92.3%	118 (96.7%)	177 (96.7%)	40%

**Table 4:** Histopathological tumor characteristics (frequencies and percentages) of invasive screen-detected and interval cancers with a malignancy score from the AI system (AI score) of 10 and AI score < 10. Tumor diameter, histologic grade, lymph node involvement, and Nottingham Prognostic Index (NPI) were calculated for invasive tumors, including no special type (NST), lobular, and other invasive

	Invasive screen-detected cancers, *n* = 754		Invasive interval cancers, *n* = 295	
	AI score = 10, *n* = 696	AI score < 10, *n* = 58	AI score = 10, *n* = 118	AI score < 10, *n* = 177
Tumor diameter, median (IQR) mm	14 (10−21)	11 (8−15)	22 (13−28)	23 (15−31)
Information not available	10	2	10	17
Histologic grade				
Grade 1	201 (29.2%)	15 (27.3%)	22 (20.6%)	21 (12.7%)
Grade 2	288 (41.9%)	24 (43.6%)	43 (40.2%)	67 (40.6%)
Grade 3	199 (28.9%)	16 (29.1%)	42 (39.3%)	77 (46.7%)
Information not available	8	3	11	12
Lymph node positive	148 (21.5%)	10 (17.9%)	47 (39.8%)	71 (40.1%)
Information not available	8	2	0	0
NPI, mean (SD)	3.6 (1.1)	3.5 (1.0)	4.0 (1.2)	4.2 (1.0)
Excellent, ≤ 2.4	161 (23.7%)	12 (22.2%)	16 (15.4%)	10 (6.3%)
Good, > 2.4 and ≤ 3.4	205 (30.2%)	20 (37.0%)	19 (18.3%)	27 (17.1%)
Moderate, > 3.4 and ≤ 5.4	271 (39.9%)	20 (37.0%)	51 (49.0%)	97 (61.4%)
Poor, > 5.4	42 (6.2%)	2 (3.7%)	18 (17.3%)	24 (15.2%)
Information not available	17	4	14	19

For interval cancers, 96.7% (118/122) with an AI score of 10 was invasive (Tables 3). The percentage was the same for cases with an AI score below 10. Median tumor diameter for those with an AI score of 10 was 22 mm (IQR: 13–28) and 23 mm (IQR: 15–31) for those with score below 10 (Table 4). A total of 39.3% (42/107) of the cases with an AI score of 10 was grade 3 tumors and 39.8% (47/118) was lymph node positive. For those with score below 10, 46.7% (77/165) was grade 3 tumors and 40.1% (71/177) was lymph node positive. Mean NPI was 4.0 (SD = 1.2) for cases with an AI score of 10 and 4.2 (SD = 1.0) for cases below 10.

### Breast density

**Fig. 2:**
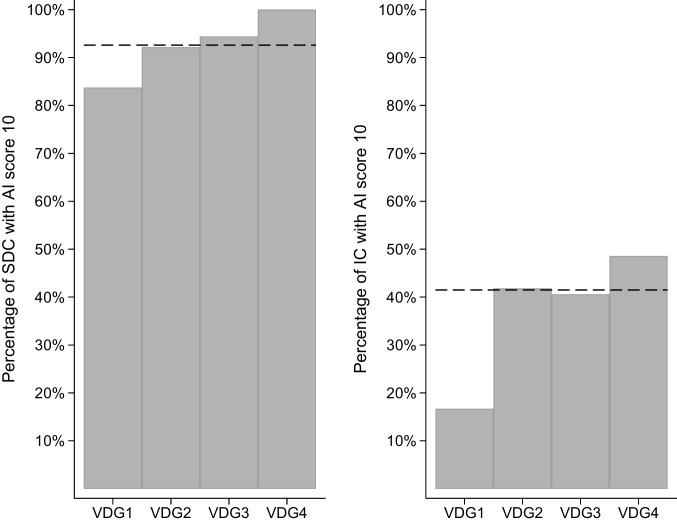
Percentage of 894 screen-detected (SDC) and 289 interval cancers (IC) with AI score 10 stratified by Volpara Density Grade (VDG, 1–4). The horizontal dotted line represents the average percentage of SDC and IC with AI score 10

In our study sample, 15% (1916/12,811) of the women were classified into VDG1, 49% (6111/12,811) into VDG2, 30% (3782/12,811) into VDG3, and 7% (891/12,811) into VDG4. Of the 59 women with screen-detected cancer and VDG4, all had an AI score of 10 (Fig. 2). Among interval cancer cases with VDG4, 48.6% (17/35) had an AI score of 10. Sensitivity for women with VDG4 was 60.6% for the first reader, 62.8% for independent double reading, and 80.9% for AI score 10. Sensitivity for women with VDG1 was 83.7% for the first reader, 93.5% for independent double reading, and 79.3% for AI score 10 (Supplementary Fig. 1).

### Prior screening examination

We found 41.9% (312/745) of the screen-detected cancer cases with a prior screening examination to have a score of 10 on the prior screening mammogram and 15.7% (117/745) to have an AI score of 9 (Table 5). A total of 17.6% (131/745) were discussed at consensus at the prior examination and 5.5% (41/745) were recalled with a negative outcome. 26.2% (64/244) of the screen-detected cancers where the prior screening mammogram had an AI score 10 were lymph node positive, compared to 17.8% (61/342) of those with an AI score below 10 (Supplementary Table 1). 5.8% of the screen-detected cancers with an AI score of 10 on the prior mammograms were classified as VDG4, compared to 4.4% for an AI score below 10.

**Table 5:** Frequencies and percentages of prior screening examinations for 754 screen-detected cancers, stratified by malignancy score of the AI system (AI score). Also, examinations discussed at consensus, recalled for further assessment, and positive interpretation by one or both radiologists, stratified by malignancy score of the AI system (AI score) are presented

AI score on prior examination	Screen-detected cancers with AI score on prior screening examination, *n* = 754				
	Prior examination for screen-detected cancers	Discussed at consensus, *n*	Recalled	Positive interpretation by one radiologist	Positive interpretation by both radiologists
1	55 (7.4%)	0 (0.0%)	0	0	0
2	26 (3.5%)	2 (1.5%)	1	1	1
3	32 (4.3%)	3 (2.3%)	1	2	1
4	28 (3.8%)	1 (0.8%)	0	1	0
5	39 (5.2%)	3 (2.3%)	0	3	0
6	37 (5.0%)	5 (3.8%)	1	5	0
7	51 (6.9%)	4 (3.1%)	2	4	0
8	48 (6.4%)	6 (4.6%)	4	2	4
9	117 (15.7%)	21 (16.0%)	8	14	7
10	312 (41.9%)	86 (65.7%)	24	62	24
Total	745 (100%)	131	41	94	37

### Pre-screen-reading triage

By excluding mammograms with AI score 1–5 from the radiologist reading stream, 54% (7475/13,896) of all examinations would be removed, including 38.7% (219/568) of the false positives, 1.6% (15/949) of the screen-detected cancers, and 24.3% (74/305) of the interval cancers (Tables 1 and 2).

## Discussion

In this retrospective study using a cancer-enriched dataset with 1254 breast cancer cases, we found that 92.7% of the screen-detected and 40.0% of the interval cancers had an AI score of 10, representing the highest risk of breast cancer. The accuracy of the AI system was comparable to one reader in an independent double-reading setting. Among cancers detected in women with the highest mammographic breast density, all screen-detected cancers and 48.6% of the interval cancers had an AI score of 10, indicating in a possible increase in sensitivity from 62.8 to 80.9%. We found 41.9% of the prior mammograms of the screen-detected cancer cases to have an AI score of 10.

The estimated sensitivity with AI threshold set at equal specificity level as the first reader suggests the performance of the AI system comparable to the radiologists and highlights the potential of implementing AI systems as the second reader in a double-reading setting. According to a systematic review from 2021, AI systems are not yet sufficiently specific to replace both radiologists in double reading, and with mixed results when comparing AI systems to a single radiologist, it is still unclear where in the interpretation process AI systems may be of most value [26]. Our results might be informative in the discussion of implementing AI in mammography screening.

The observed difference in histopathological tumor characteristics and NPI between screen-detected and interval cancers as two groups independent of AI scores corresponded well with results from other studies [8, 9]. NPI is a clinically relevant and internationally validated scoring system used to determine the long-term prognosis following surgery for breast cancer patients [23, 27–29]. The AI system scored 10 on a considerable number of interval cancers with high NPI, but the potential effect on breast cancer mortality needs a proper investigation in prospective studies before any conclusion can be drawn. Results on tumor size, histologic grade, lymph node involvement, and NPI indicated less favorable tumor characteristics for interval cancers with an AI score below 10 compared to those for interval cancers with an AI score of 10, which might indicate that these were true interval cancers, associated with rapid growth and aggressive potential.

Women with mammographic extremely dense breast have 3–6 times higher risk of developing breast cancer compared to women with fatty breast and do also represent a challenge for radiologists due to the masking effect of the dense tissue on tumors [7, 30, 31]. Due to an increased risk of advanced disease at diagnosis for women with extremely dense breast, The European Society of Breast Imaging (EUSOBI) recommend offering these women screening with MRI every 2–4 years [7]. However, EUSOBI acknowledges that this may not be feasible with current radiological resources [7]. We found that AI identified a large proportion of cancers in extremely dense breasts (VDG4); all screen-detected cancers and almost half of the interval cancers among women with the highest density score had an AI score of 10. If similar results can be obtained in a prospective screening setting, increased cancer detection among women with extremely dense breasts might be possible without adding supplementary modalities. In a Danish study, the same AI system as we used maintained persistently high area under the receiver operating characteristic curve (AUC) values with increasing breast density [14], as opposed to radiologists. The high sensitivity of the AI system in dense breasts needs to be further investigated and should be considered in the triaging in future prospective studies.

A substantial percentage of the screen-detected cancers were shown to have an AI score of 10 on the mammogram preceding the mammogram where the cancer was diagnosed, and these cases had a higher NPI and were more often lymph node positive compared to cancer cases with an AI score below 10. Although this group had a slight overweight of extremely dense breasts, our findings indicate a potential for earlier detection of relevant cancers with the aid of AI systems. As far as we know, no study to date has used AI to examine prior mammograms for women with screen-detected cancers, but our observations corresponds well with current knowledge based on retrospective consensus-based informed review studies classifying 20–30% of the screen-detected cancers as missed cancers [10, 11]. Interestingly, 86 of the screen-detected cancers with AI score 10 were discussed at consensus of the preceding round, and 24 women had a “false-positive” screening result, recalled for further examinations with negative outcome. Our observations associates well with current knowledge: women discussed at consensus, either dismissed or recalled, have an increased risk of cancer the consecutive screening round [32]. A review of the mammograms would be required to conclude according to these observations.

Our results support results from other studies indicating beneficial effects of using AI as a second reader or as a triaging tool in mammography screening [14, 16, 17, 21, 22]. Both approaches, either alone or combined, would significantly reduce the reading volume for radiologists. However, we must be aware of the lack of evidence as to how AI could affect the consensus process in a real screening setting. Even if the AI systems were able to achieve improved sensitivity and specificity compared to radiologists, the effect on consensus, actual callback rate, and ultimately the mortality rate are yet unknown and require specific prospective studies. The performance of AI systems in a real-time clinical setting is reliant on the calibration of the AI raw score threshold, depending on the AI system’s role in the specific screening setting, e.g., triage, selection for consensus, or direct recall. As an example, selecting all women with an examination with an AI score of 10 for direct recall without further adjustment of the AI threshold would yield an unacceptably high recall rate around 10%, 2–4 times higher than what is currently normal in BreastScreen Norway [15].

Strengths in our study are the large number of cancer cases, automated measures of mammographic density, and that image data was merged with complete screening data from the Cancer Registry of Norway, a data resource that is close to 100% complete for breast cancer [33]. Limitations were related to the cancer enrichment, only including examinations from GE Senographe Essential machines and only including examinations from one screening area. Due to issues regarding external validity in enriched datasets, estimated sensitivity and specificity were appraised as comparison tools only, and not as absolute values. Neither AUC values, positive predictive values, or negative predictive values were calculated. For the same reason, estimated workload reduction and avoidable false positives in a pre-screen-reading triage setting are likely too high. A dataset representing an authentic screening population is preferred over an cancer-enriched dataset.

In conclusion, our study based on retrospective cancer-enriched data and one AI system indicated promising results in breast cancer screening with standard digital mammography, where AI systems may be effective as a second reader or a triaging tool. The sensitivity for detecting cancers in women with extremely dense breasts was higher for the AI system compared to that for the radiologists. Further, 40% of the screen-detected cancer had a score of 10 at prior screening exam, indicating a potential for earlier detection. Further, our results indicated that the screening volume for the radiologists might be reduced without reducing the cancer detection.

## Supplementary Information

Below is the link to the electronic supplementary material.

Supplementary file1 (PDF 95 KB)

## Abbreviations

AI: Artificial intelligence
DCIS: Ductal carcinoma in situ
EUSOBI: European Society of Breast Imaging
NPI: Nottingham Prognostic Index
VDG: Volpara Density Grade

## Declarations

### Guarantor

The scientific guarantor of this publication is Solveig Hofvind.

### Statistics and biometry

One of the authors has significant statistical expertise.

### Methodology

• Retrospective

• Registry study

• Performed at one institution
