# Supplementary material for: Artificial intelligence in BreastScreen Norway: a retrospective analysis of a cancer-enriched sample including 1254 breast cancer cases
Source: Eur Radiol. 2023 Mar 14;33(5):3735–43. doi: 10.1007/s00330-023-09461-y (PMC10121532; doi:10.1007/s00330-023-09461-y)

Supplementary Table 1. Histopathological tumor characteristics (frequencies and percentages) of cancers with a malignancy score by the AI system (AI score) of 10 and AI score<10 at prior screening. Tumor diameter, histologic grade, lymph node involvement and Nottingham prognostic index (NPI) were calculated for invasive tumors, including no special type (NST), lobular and other invasive.

	Screen-detected cancers, n=745	
	AI score=10, n=312	AI score<10, n=433
Ductal carcinoma in situ	68 (21.8%)	86 (19.9%)
Invasive carcinoma NST	210 (67.3%)	305 (70.4%)
Invasive lobular carcinoma	25 (8.0%)	23 (5.3%)
Other invasive	9 (2.9%)	19 (4.4%)
Invasive tumors	244 (78.2%)	347 (80.1%)
Tumor diameter, median (IQR) mm	15 (12-21)	12 (9-18)
Information not available	3	5
Histologic grade		
Grade 1	79 (32.5%)	95 (28.0%)
Grade 2	96 (39.5%)	142 (41.9%)
Grade 3	68 (28.0%)	102 (30.1%)
Information not available	1	8
Lymph node positive	64 (26.2%)	61 (17.8%)
Information not available	0	5
NPI, mean (SD)	3.6 (1.1)	3.5 (1.0)
Excellent, ≤2.4	59 (24.5%)	82 (24.5%)
Good, >2.4 and ≤3.4	62 (25.7%)	114 (34.0%)
Moderate, >3.4 and ≤5.4	103 (42.7%)	121 (36.1%)
Poor, >5.4	17 (7.1%)	18 (5.4%)
Information not available	3	12

Supplementary Figure 1. Sensitivity for the AI system (AI score 10) and the first reader, stratified by Volpara Density Grade (VDG, 1-4).

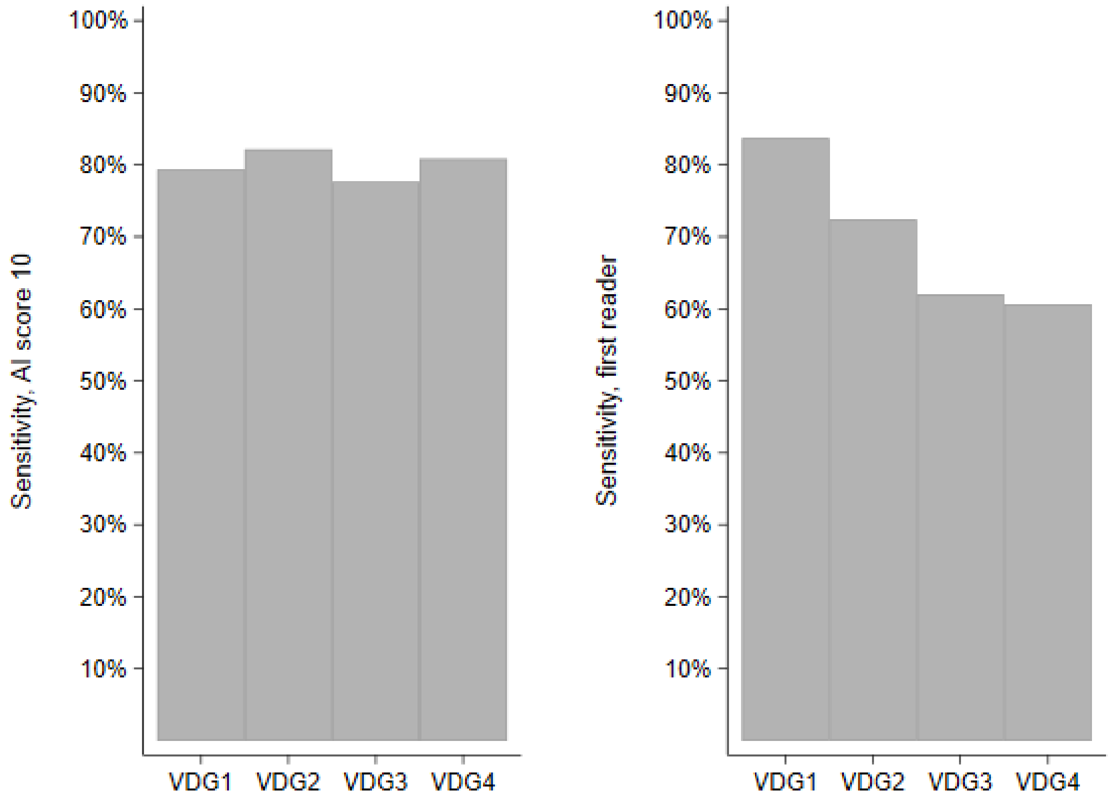

Supplement: Supplementary file 1 — Supplementary file1 (PDF 95 KB) [file 330_2023_9461_MOESM1_ESM.pdf]
